# Intravesical hyaluronic acid and chondroitin sulfate for recurrent urinary tract infections: systematic review and meta-analysis

**DOI:** 10.1007/s00192-017-3508-z

**Published:** 2017-11-27

**Authors:** Jonathan Charles Goddard, Dick A. W. Janssen

**Affiliations:** 1 Department of Urology, Leicester General Hospital, Leicester, UK; 2 Department of Urology, Radboud University Medical Center, Geert Grootteplein Zuid 10, 6525 Nijmegen, GA Netherlands

**Keywords:** Chondroitin sulfate, Female, Hyaluronic acid, Urinary tract infections

## Abstract

**Introduction and hypothesis:**

The objective was to assess the efficacy of intravesical hyaluronic acid (HA) and chondroitin sulfate (CS), alone or in combination, for recurrent urinary tract infections (RUTIs) in adult female patients using a systematic review and meta-analysis.

**Methods:**

English-language articles were obtained from the MEDLINE, Embase, and Cochrane databases through November 2016, by manual searching and cross-referencing. Randomized and nonrandomized trials of adult female patients with a documented history of RUTIs who received HA, CS or HA plus CS were included. The random effects model was applied to all pooled analyses. Risk of bias was assessed for individual studies and across studies.

**Results:**

Two randomized (*n* = 85) and six nonrandomized (*n* = 715) studies met the inclusion criteria. These studies assessed HA ± CS; studies of CS alone were not identified in the search. HA ± CS decreased the UTI rate per patient-year (pooled mean difference [MD] –2.56; 95% confidence interval [CI] –3.86, −1.26; *p* < 0.001) and increased the time to first UTI recurrence (pooled MD 130.05 days; 95% CI 5.84, 254.26; *p* = 0.04). There was heterogeneity in most outcomes considered, and publication bias in many studies. The standard of trial reporting was low. The patient population size, and the number of studies included, were small.

**Conclusions:**

HA ± CS appears to reduce the rate of UTI and increase the time to recurrence in women with RUTI. As randomized controlled studies are available only for HA plus CS, the quality of evidence is higher for the combination than for HA alone.

**Electronic supplementary material:**

The online version of this article (10.1007/s00192-017-3508-z) contains supplementary material, which is available to authorized users

## Introduction

Urinary tract infections (UTIs) occur more frequently in women than in men, and are a major female healthcare concern, with an annual incidence of 30 per 1,000 people [1]. Over a lifetime, an estimated 40–50% of women experience at least one episode of UTI [2]. UTIs have a tendency to recur, with the risk of a second infection being 24–27% within 6 months of the initial episode [3, 4], and approximately 70% within 12 months [5, 6]. According to the European Association of Urology guidelines, recurrent UTI (RUTI) is defined as the occurrence of at least three episodes of uncomplicated infection, documented by positive urine culture (>10^3^ colony-forming units/mL [cfu]) in the previous 12 months [7].

Although the specific strategy for patient care depends on individual clinical characteristics (e.g., the number of recurrences per year), risk factors, and preferences, RUTIs are commonly managed with intermittent or prolonged antibiotics therapy [7]. However, conventional prophylaxis does not always appear to provide satisfactory results. There is also the important issue of antibiotics resistance. Antibiotics resistance is a growing problem worldwide, with consumption of antibiotics being a major risk factor for the development of resistance [8, 9]. Some analyses have shown that higher rates of resistance are associated with longer treatment duration and the consumption of multiple courses of antibiotics [9]. In isolates from UTIs, varying degrees of resistance to multiple antibiotics are commonly reported [10–15], and those studies that have investigated resistance rates over time have reported that antibiotic resistance rates are increasing [11, 14, 15]. For these reasons, there has been a growing interest in finding alternative therapeutic and prophylactic drugs for RUTIs.

Another strategy for the management of RUTIs is based on the re-establishment of the GAG layer of the bladder epithelium, particularly with intravesical instillations of hyaluronic acid (HA), alone or in combination with chondroitin sulfate (CS) [16–18]. The efficacy of this therapy in patients with RUTIs has been assessed in various randomized and nonrandomized studies [17–24] and evaluated in systematic reviews or meta-analyses [25, 26], which confirm the positive effect of reducing the frequency of RUTIs.

The most recently conducted meta-analysis of the efficacy of HA (with or without CS) in the prevention of RUTIs was published in 2013 [26]. An updated summary of the current clinical evidence regarding the efficacy of therapy with HA and CS, alone or in combination, would therefore be useful. As such, a meta-analysis and systematic review of all available clinical data (including randomized controlled trials and nonrandomized studies) regarding the efficacy of HA and CS in patients with RUTIs was conducted. The primary objective of this meta-analysis and systematic review was to investigate whether HA and CS, alone or in combination, are more effective than other prophylactic treatments or placebo in reducing the occurrence of RUTIs in adult female patients.

## Materials and methods

This systematic review and meta-analysis complies with the Preferred Reporting Items for Systematic Reviews and Meta-Analyses Guidelines [27]. A review protocol was specified in advance, but not prospectively registered.

### Study eligibility criteria

The systematic review included randomized (both open-label and double-blind design) and nonrandomized studies investigating intravesical instillation of HA, CS, or the combination for the prevention of RUTIs in adult female patients. The review was limited to English-language publications; however, there were no publication date or publication status restrictions (congress abstracts were also considered). Study patient populations were adult women with RUTIs. Studies that involved both women and men were only included if the data relating to female participants were presented separately. Studies were required to include an intervention of intravesical instillation of HA and/or CS and a control group, defined as placebo, standard of care prophylaxis, or retrospective patient review. The primary outcomes were mean rate of UTI episodes per patient-year and mean time to first UTI recurrence (in days). The secondary outcomes were the number of patients with UTI recurrence, number of 3-day voids, pelvic pain and urgency/frequency (PUF) total, and symptom scale scores and quality of life measures (VAS, SF-36, and KHQ). Studies were included if they reported at least one of the above outcomes.

### Search strategy

A literature search was performed using MEDLINE (accessed by PubMed), Embase (1947 to present), and the Cochrane Central Register of Controlled Trials to identify relevant studies published in English up to 9 November 2016. The following search terms were used to search all databases and trial registers: urinary tract infections; bacteriuria; bacilluria; cystitis; cystitides; urinary; bladder; urine; urologic; ureteral; infection*; inflammation; pyelonephritis; urosepsis; UTI; RUTI; CAUTI; hyaluronic acid; hyaluron*; chondroitin sulfates; chondroitin*; condroitin; sulfate; sulfate; Cystistat; Gepan; Uracyst; Hyacyst; Hyachon Duo; Instillamed. The strategy specifically focused on studies in human women, whereas studies in animals, men, adolescents and children, and the elderly were filtered out during the literature search; full search strategies for each database used are available in the Supplementary material. The search was additionally supplemented from the reference lists of the systematic reviews identified [16, 23, 25, 28–31] and by a manual search of the reference lists of the articles.

### Study selection and data collection

Titles and abstracts of retrieved articles were independently screened for eligibility by two external reviewers. When abstracts had insufficient information for analysis of the intervention or methodology regarding the inclusion and exclusion criteria defined in this review, a full-text review was necessary. Subsequently, the two reviewers evaluated full-text articles to determine study eligibility. In the event that a disagreement occurred between the reviewers, a discussion took place and the final decision was made only once consensus had been reached. A list of the excluded articles is available in the Supplementary material.

Data extraction was conducted using an ad hoc extraction form with the following fields: author; year of publication; site; type of study; intervention and comparator; length of follow-up; number of participants by group; and outcome. Where data were reported as median and range (minimum, maximum), a corresponding mean and SD was estimated using a previously published formula; [32] when results were reported as median and interquartile range, the corresponding authors were contacted to obtain more information. The corresponding authors were not contacted to obtain other outcome data not included in the published report.

### Risk of bias assessment

The risk of bias within each individual study was evaluated using different items, depending on the study type. For randomized trials, the risk of bias was assessed using the following items: adequate sequence generation; allocation concealment; blinding of outcomes (participants and personnel); blinding of outcome (assessors); description of losses and exclusions; use of intention-to-treat analysis; and incompleteness of outcome data. Each category was judged using the Cochrane risk of bias tool [33]. Nonrandomized trials were evaluated using the following items from the Risk of Bias in Nonrandomized Studies of Interventions Score [34]: confounding bias; selection bias; classification bias; bias due to deviation from intended interventions; bias due to missing data; bias in the measurement of outcome; and bias due to the selection of reported results. In some cases (particularly nonrandomized trials), there was insufficient or no information available to assess whether or not an important risk of bias existed. No study was excluded from review if judged “high risk of bias”, but the findings from any such trial were regarded with increased caution.

To assess the risk of bias across studies, a funnel plot of trial-standardized MD by standard error was constructed to evaluate the possibility of publication bias [35]. The symmetry of the plot was evaluated both visually and formally using Egger’s test.

### Data synthesis and analysis

The primary measures of treatment effect were mean rate of UTI episodes per patient-year and mean time to first UTI recurrence (in days); the MD in each outcome was the association measure. Secondary outcome measures were number of 3-day voids, PUF total and symptom scores, SF-36 score (with MD as the association measure), and the proportion of patients free from UTIs at the end of follow-up (with RR as the association measure).

The random-effects model was used for pooling data from the primary studies, as clinical heterogeneity was expected. Statistical heterogeneity of the treatment effect among studies was assessed using the inconsistency I^2^ test, in which values >30% were considered to be indicative of high heterogeneity. Between-study heterogeneity was performed using the Chi-squared test. A *p* value of <0.05 was considered statistically significant.

Sensitivity and subgroup analyses were pre-specified for primary outcomes. In the sensitivity analyses, the association measure was examined by omitting studies individually. Subgroup analyses aimed to assess whether there was a difference in the results of the association measure between nonrandomized versus randomized studies, noncontrolled versus controlled studies, and studies with different treatment intervention (HA vs HA plus CS).

Statistical analysis was performed using R Statistical Software (Foundation for Statistical Computing, Austria).

## Results

### Study selection

**Fig. 1:**
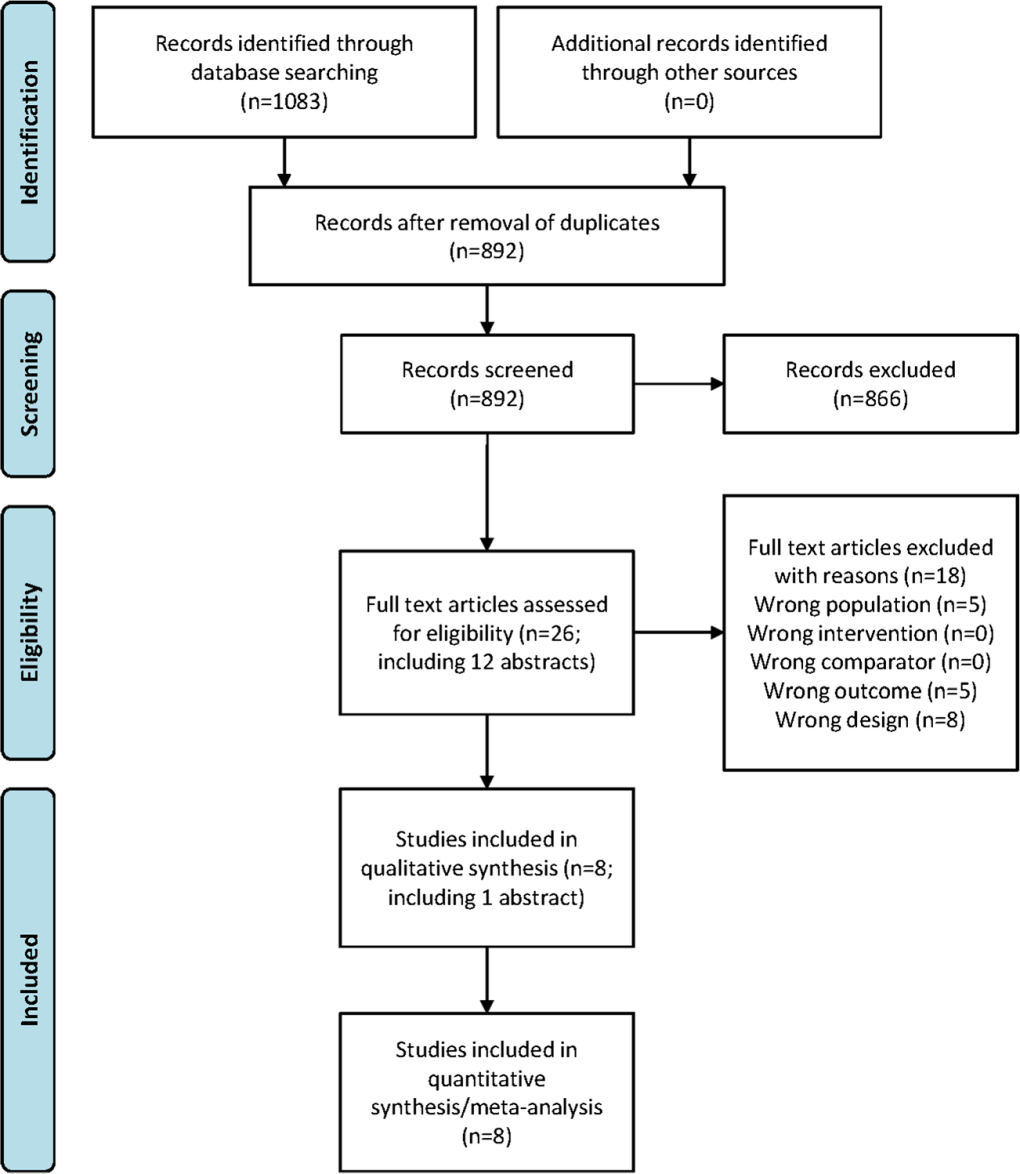
Flow diagram of the study search and selection process for systematic review and meta-analysis

A flow diagram of the study search and selection is shown in Fig. 1. The database search resulted in 1,083 articles (305 from PubMed, 610 from Embase, and 168 from the Cochrane trial register), of which 892 were not duplicated. Of these, 866 were excluded after reviewing the title and/or abstract as they did not appear to meet the study inclusion criteria. The full text of the remaining 14 articles and 12 abstracts (total 26 citations) were assessed for eligibility in more detail. Eighteen articles did not meet the inclusion criteria and were excluded. Eight articles met the eligibility criteria and were included in the systematic review and meta-analysis.

### Study characteristics

The main characteristics of the included studies are summarized in Table 1. Among the 8 studies selected for the review, 1 was published as an abstract only. All studies took place in Europe, and 3 were multicenter trials. Some studies had received partial industry sponsorship. Two studies were randomized controlled trials [22, 23] and 6 studies were of nonrandomized design [17–21, 24]. The duration of treatment intervention ranged from 2 to 6 months and the duration of total follow-up ranged from 12 to 18 months.

**Table 1:** Characteristics of the studies of intravesical instillations of hyaluronic acid included (with or without chondroitin sulfate) and key efficacy and safety results

Reference	Study design	Patients	Intervention	Comparator	Primary outcome(s)	Key efficacy results	Safety
Constantinides et al. [18]	Nonrandomized, noncontrolled study of HA in the prevention of RUTIs	Women aged 18–45 years (mean age 35 years) with a history of RUTIs (*n* = 40)	HA (Cystistat^®^) 40 mg in 50 mL of PBS once weekly for 4 weeks, then once monthly for 4 months, followed for a further 7 months	Retrospective review of patient records from before HA treatment (mean duration 15.8 months)	Mean rate of UTI per patient-year; median time to first recurrence	Mean rate of UTI per patient-year before HA 4.3 vs after HA 0.3 (*p* < 0.001); median time to recurrence before HA 96 days vs after HA 498 days (*p* < 0.001)	No serious AEs: 9 cases of mild bladder irritation after instillation, 3 patients required anti-inflammatory medication
Lipovac et al. [17]	Nonrandomized, noncontrolled study of HA in the prevention of RUTIs	Women aged 17–34 years (mean age 28 years) with a history of RUTIs (*n* = 20)	HA (Cystistat^®^) 40 mg in 50 mL of PBS once weekly for 4 weeks, then once monthly for 5 months, followed for a further 6 months	Retrospective review of patient records from before HA treatment (mean duration 36 weeks)	Mean rate of UTI per patient-year; mean time to first recurrence	Mean rate of UTI per patient-year before HA 4.99 vs after HA 0.56 (*p* < 0.001); mean time to recurrence before HA 76.7 days vs after HA 178.3 days (*p* < 0.001)	No serious AEs: 18 cases of mild to moderate pain during instillation, 6 cases of cramping/burning, 3 required anti-inflammatory medication
Damiano et al. [22]	Randomized, double-blind, placebo-controlled study of HA-CS in the prevention of RUTIs	Women (mean age 35 years) with a history of RUTIs (*n* = 57)	50 mL of HA 1.6% plus CS 2.0% solution (Ialuril^®^) once weekly for 4 weeks, then once monthly for 5 months, followed for 12 months in total (*n* = 28)	50-mL placebo (saline) administered with the same schedule, followed for 12 months in total (*n* = 29)	Mean number of UTIs per patient-year	Mean number of UTIs per patient-year in HA-CS group 0.67 vs in placebo group 4.19 (*p* < 0.001)	No serious AEs: 3 cases of moderate urinary storage symptoms with HA-CS (vs none with placebo), 1 patient required anti-inflammatory medication
Centemero et al. [19]	Nonrandomized, noncontrolled study of HA in the prevention of RUTIs	Women aged 25–48 years (mean age 37 years) with a history of RUTIs (*n* = 48)	HA (Cystistat^®^) 40 mg in 50 mL of PBS once weekly for 8 weeks, followed for 18 months in total	Retrospective review of patient records from before HA treatment	Mean time to UTI recurrence	Mean time to UTI recurrence before HA 39.85 days vs after HA 190.64 days (*p* < 0.001)	No serious AEs
De Vita and Giordano. [23]	Randomized, controlled study of HA-CS in the prevention of RUTIs	Women (mean age 60 years) with a history of RUTIs (*n* = 28)	HA 800 mg plus CS 1 g (Ialuril^®^) in 50 mL of saline solution once weekly for 4 weeks, then once every 2 weeks for 4 weeks, followed for 12 months in total (*n* = 12)	SMX 200 mg plus TMP 40 mg once weekly for 6 weeks, followed for 12 months in total (*n* = 14)	Mean number of UTI after 2 and 12 months’ follow-up	Mean number of UTIs after 2 months in the HA-CS group 3 vs in the control group 2.9; after 12 months in the HA-CS group 1 vs in the control group 2.3 (*p* = 0.02)	No AEs
Cicione et al. [21]	Multicenter, retrospective, nonrandomized, noncontrolled (observational) study of HA-CS in the prevention of RUTIs	Women (mean age 53 years) with a history of RUTIs (*n* = 157)	50 mL of HA 1.6% plus CS 2% (Ialuril^®^) once weekly for 4 weeks, then once monthly for 5 months, followed for up to 24 months	Retrospective review of patient records before HA-CS treatment (12 months)	Mean number of UTIs per patient-year; mean time to UTI recurrence	Mean number of UTIs per patient-year before HA-CS 4.13 vs after HA-CS 0.44 (*p* = 0.01); mean time to UTI recurrence before HA-CS 94.8 days vs after HA-CS 178.4 days (*p* = 0.01)	10 cases of moderate urinary storage symptoms (moderate pain after instillation), 1 patient required medication for symptom relief
Gugliotta et al. [24]	Multicenter, retrospective, cohort study of HA-CS in the prevention of RUTIs	Women (mean age 38 years) with a history of RUTIs (*n* = 174)	HA 1.6% plus CS 2% (Ialuril^®^) in 50 mL of water plus calcium chloride once weekly for 4 weeks, then once monthly for 4 months, followed for a further 12 months (*n* = 98)	SMX 200 mg plus TMP 40 mg once daily for 6 weeks, followed for a further 12 months (*n* = 76)	Total number of UTIs recorded over 12 months; percentage of patients UTI-free at 12 months	At 12 months, total number of UTIs in the HA-CS group 69 vs in the control group 109; percentage of patients UTI-free, in the HA-CS group 36.7% vs in the control group 21.0% (*p* = 0.03)	No serious AEs; 78% of patients reported mild/moderate pain or burning during instillation, 22 patients required anti-inflammatory medication
Ciani et al. [20]	Multicenter, retrospective, case–control study of HA-CS in the prevention of RUTIs	Women aged 18–75 years (mean age 53 years) with a history of RUTIs (*n* = 276)	HA 1.6% plus CS 2.0% (Ialuril^®^) once weekly for 4 weeks, then once every 2 weeks for 4 weeks and once monthly thereafter (different patterns used; a maximum of 7 installations; *n* = 181)	Standard of care prophylaxis (antimicrobial, immunoactive, probiotics, cranberry or a combination; *n* = 95)	Objective UTI recurrence within 12 months of treatment	Bacteriologically confirmed UTI in the HA-CS group 55.7% of patients vs in control group 62.1% of patients (*p* = 0.313)	Not reported

*AE* adverse event,* CS* chondroitin sulfate,* HA* hyaluronic acid,* PBS* phosphate-buffered saline,* RUTI* recurrent urinary tract infection,* SMX* sulfamethoxazole,* TMP* trimethoprim,* UTI* urinary tract infection

The eight studies included a total of 800 patients: of these, 478 patients received intravesical instillations of HA plus CS (Ialuril^®^, IBSA), 108 patients received intravesical instillations of HA (Cystistat^®^, Bioniche), and 214 received comparator therapy with placebo, oral sulfamethoxazole plus trimethoprim prophylaxis or other standard of care prophylaxis. In 265 patients, response to HA or HA plus CS treatment was compared with a retrospective assessment of UTI recurrence before treatment. In all studies, the main inclusion criterion was a documented history of RUTI, defined as at least three episodes of uncomplicated UTI with clinical symptoms and/or a positive culture (>10^3^ cfu/mL) in the past 12 months, in adult female patients. In all trials, the primary outcome(s) assessed were the mean UTI rate per patient-year and/or the mean or median time to first UTI recurrence (in days). Secondary outcomes included the number of 3-day voids, PUF total and symptom scores, SF-36 scores, and the percentage of patients who were UTI-free during follow-up.

### Risk of bias within studies

The potential sources of bias within each study were assessed. In general, the standard of trial reporting was poor, particularly in the nonrandomized trials. There were insufficient data to evaluate potential bias in some studies. The extent of methodological bias in this group of studies is therefore difficult to accurately determine, as shown by the high frequency of “unclear” judgments in both randomized (Table S1) and nonrandomized (Table S2) trials.

### Results of individual studies

**Fig. 2:**
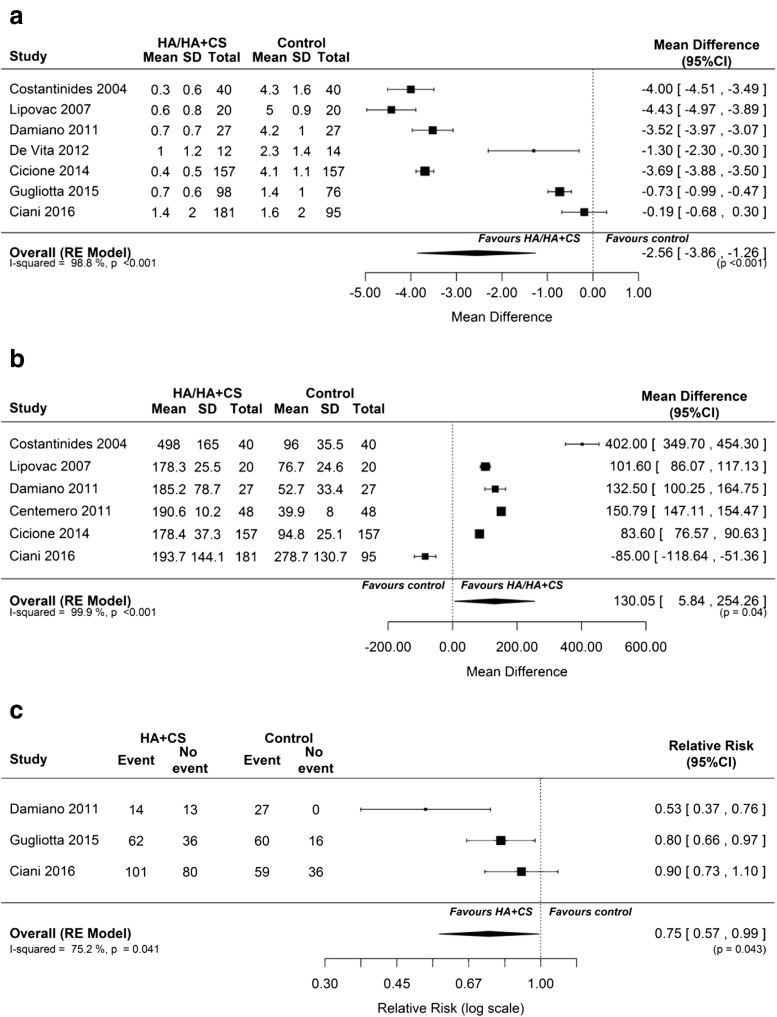
The effect of hyaluronic acid/hyaluronic acid plus chondroitin sulfate versus controls on **a** mean urinary tract infection (UTI) rate per patient-year, **b** time to first UTI recurrence (in days), and **c** percentage of patients with UTI recurrence during follow-up.* CI* confidence interval,* CS* chondroitin sulfate,* HA* hyaluronic acid,* RE* random effects,* SD* standard deviation

Overall, the individual studies showed a positive treatment effect with HA or HA plus CS for the two primary outcomes, with statistically significant differences from comparator therapy observed in most studies (Table 1). When compared with control treatment (i.e., placebo, standard of care prophylaxis or a retrospective review of patient history), HA, with or without CS, was associated with a significantly lower mean UTI rate per patient-year (MD –2.56; 95% CI –3.86, −1.26; *p* < 0.001; Fig. 2a) and a significantly longer time to UTI recurrence (MD 130.05 days; 95% CI 5.84, 254.26; *p* = 0.04; Fig. 2b). There was evidence of heterogeneity for both outcomes (I^2^ = 98.8%; *p* < 0.001 for heterogeneity and I^2^ = 99.9%;* p* < 0.001 for heterogeneity respectively), and the random effects model was used for these analyses.

**Fig. 3:**
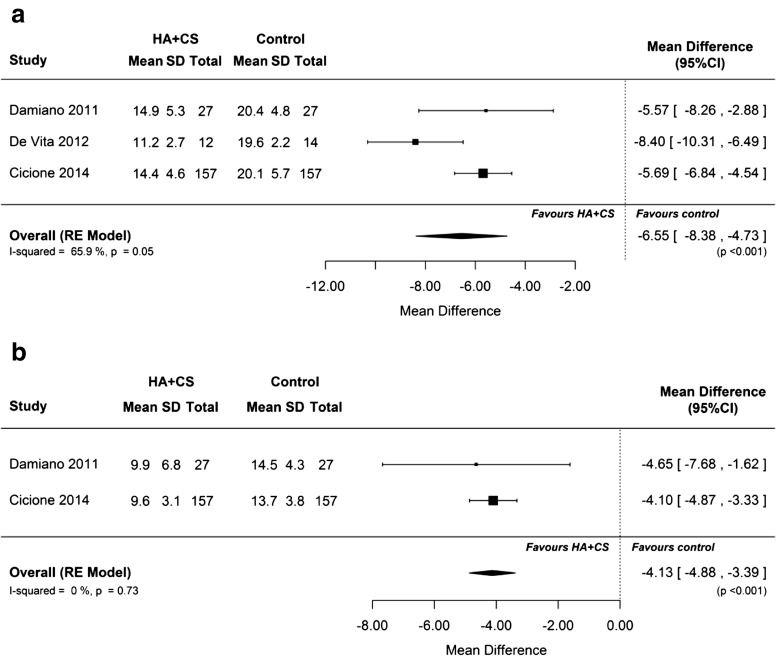
The effect of hyaluronic acid plus chondroitin sulfate versus control on pelvic pain and urgency/frequency (PUF) **a** total and **b** symptom scores. PUF score lower and upper limits: 0–36

Among the secondary outcomes, HA plus CS was associated with significantly greater mean reductions in PUF total and symptom scores than control treatment (*p* < 0.001; Fig. 3). The percentage of patients with UTI recurrence during follow-up was also lower with HA plus CS than in the control group (RR 0.75; 95% CI 0.57, 0.99; *p* = 0.043; Fig. 2c). However, the number of 3-day voids and SF-36 outcomes did not show significant differences between the HA plus CS and control groups (Figs. S1A and S1B). The random-effects model was applied in each of above analyses.

### Risk of bias across studies

**Fig. 4:**
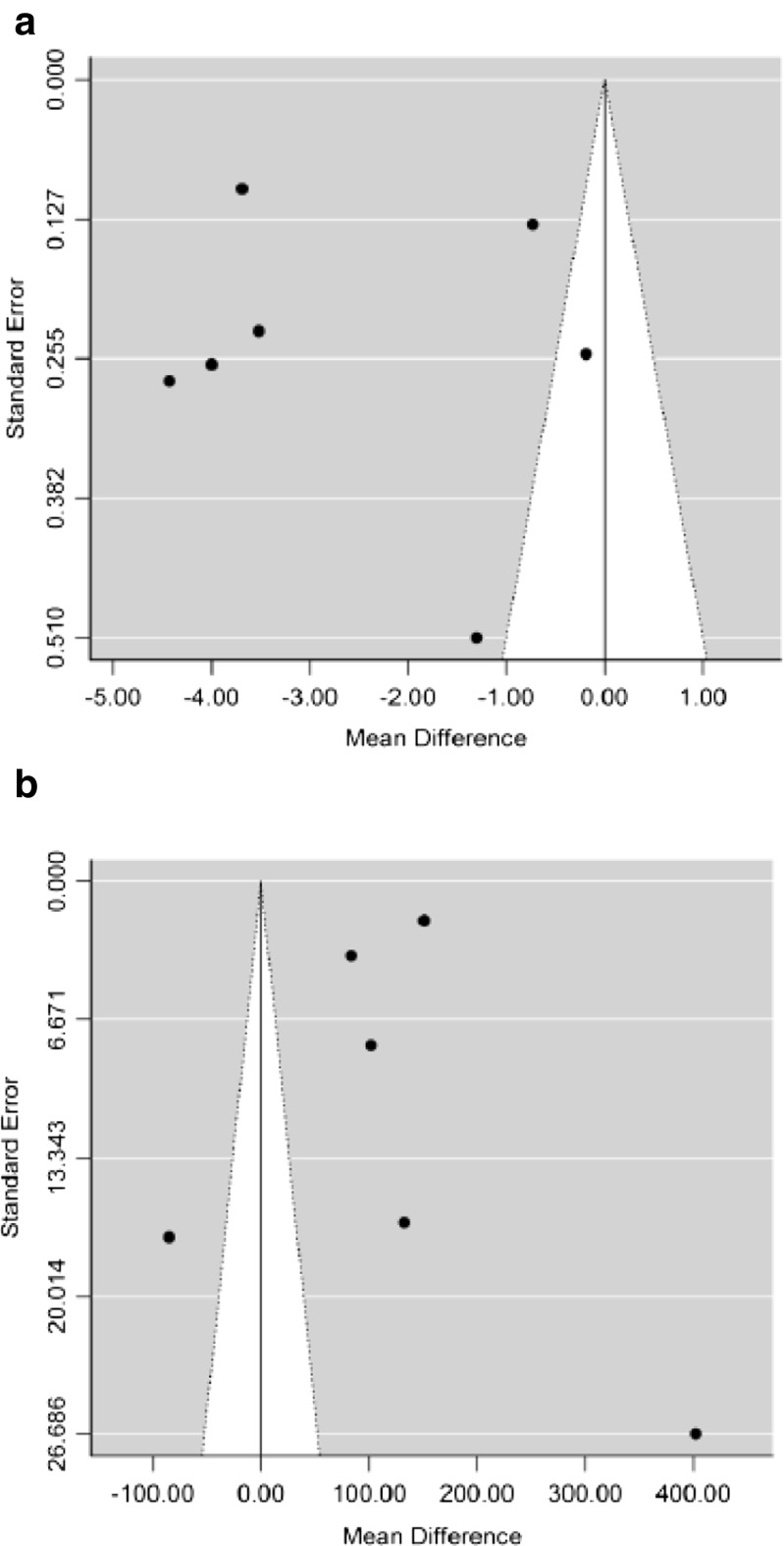
Risk of bias for **a** mean UTI rate per patient-year and **b** time to first UTI recurrence

Funnel plots for both primary outcomes show evidence for asymmetry (Fig. 4). For the mean UTI rate per patient-year outcome, all published studies appear on the left of the plot (indicating a better impact of treatment), which suggests that some studies are missing from the right (a negative impact of treatment; Fig. 4a). For the time to first UTI recurrence outcome, most studies appear on the right of the plot (indicating a better impact of treatment), which means that some studies are possibly missing from the left (a negative impact of treatment; Fig. 4b. However, the low number of studies does not allow for reliable interpretation of the funnel plots or Egger’s tests, and thus cannot be considered definitive with regard to the assessment of bias.

### Additional analyses

Subgroup analyses showed a similar treatment effect in nonrandomized (*n* = 6) versus randomized (*n* = 2) studies (Fig. S2). The treatment effect was greater in noncontrolled (*n* = 4) than controlled (*n* = 4) studies, especially with regard to the mean UTI rate per patient-year (MD –3.98 [95% CI –4.41, −3.55] vs MD –1.44 [95% CI –2.90, 0.02]; Fig. S3). When comparing those studies that reported the use of antibiotics in the control group and those that did not, a considerably greater reduction in the mean UTI rate per patient-year was observed in the latter (Damiano et al. [22]: MD –3.52 [95% CI –3.97, −3.07]) compared with the former (De Vita and Giordano [23]: –1.30 [95% CI –2.30, −0.30]; Gugliotta et al. [24]: –0.73 [95% CI –0.99, −0.47]; Ciani et al. [20]: –0.19 [95% CI –0.68, 0.30]; Fig. S3). Greater reductions in the mean UTI rate per patient-year and a longer time to first UTI recurrence were observed in HA (*n* = 3) compared with HA plus CS (*n* = 5) studies (Fig. S4), most likely driven by the results of Ciani and colleagues [20].

In sensitivity analyses, in which the studies were individually omitted from the meta-analysis, the MD remained generally similar and always statistically significant with regard to mean UTI rate per patent-year outcome, ranging from −2.25 (95% CI: −3.61 to −0.89) to −2.96 (95% CI –4.19, −1.73; Table S3). Data regarding the time to first UTI recurrence outcome seem to be less robust; if any of the studies were removed (other than the study by Ciani et al. [20]), the MD showed no statistical significance (Table S3).

## Discussion

This systematic review and meta-analysis examines all the available evidence captured from existing nonrandomized and randomized clinical studies on the effectiveness of HA or HA plus CS for the prevention of RUTIs in adult female patients. Of note, this meta-analysis did not include any studies investigating CS alone, because studies using CS 0.2% or 2.0% alone were not identified using the search strategy employed in this analysis. In the eight trials included in this review (800 patients), there was a mean reduction in UTI episodes of ≈2.6 episodes per patient-year (Fig. 2a) and a mean increase in the time to first UTI recurrence of ≈130 days (Fig. 2b) in female patients treated with HA or HA plus CS compared with placebo, standard of care prophylaxis or retrospective review of UTI recurrence. HA plus CS was also associated with mean reductions in PUF total scores of ≈6.5 points (Fig. 3a) and symptom scores of ≈4 points (Fig. 3b), and a 25% reduction in the risk of UTI recurrence (Fig. 2c) compared with the control group.

Of note, a considerably greater reduction in the mean UTI rate per patient-year was observed in the study by Damiano et al. [22] compared with other controlled studies, namely, De Vita and Giordano [23], Gugliotta et al. [24], and Ciani et al. [20]. This is likely explained by the use of placebo as the comparator in the study by Damiano et al. [22], whereas in the other controlled studies, antibiotics therapy was used as the comparator. Therefore, it is reasonable to suggest that the reduction in the rate of UTIs of ≈2.6 episodes per patient-year represents a conservative estimate of the efficacy of HA in combination with CS.

In addition to treatment efficacy, the safety of HA therapy must be considered when interpreting the results of this meta-analysis. The method of administration (intravesical instillation) is more invasive than other standard of care options (usually oral), and can therefore be more painful. Among the studies where adverse event data were reported, no serious adverse events occurred; however, some cases of mild bladder irritation and mild to moderate pain, cramping or burning after instillation were reported (Table 1). This type of nonserious adverse event was reported by 116 patients across all studies, with 29 requiring anti-inflammatory medication to relieve symptoms. All these patients were in the study treatment group (Table 1).

The main limitation of this meta-analysis was the quality of the primary studies, in which the methods and reporting of data were generally poor. The treatment schedules varied across studies, with most studies adopting a treatment schedule of one instillation every week for 4 weeks (7 out of 8 studies), followed by a monthly instillation for 4–5 months (5 out of 8 studies). The most common treatment duration was 6 months, and it may be that a treatment duration of 12 months would improve treatment efficacy; however, more data are required before the best strategy for use in clinical practice can be determined. An additional confounding factor may be that there were differences in the control treatment group, with some studies using antibiotics as the comparator and other studies using placebo, which led to a much greater heterogeneity in the control arm outcome compared with the heterogeneity in the treatment arms. However, because differences in methodology were expected, the random effects model was used for pooling data and the analyses were stratified on the basis of study design (randomized vs non-randomized, controlled vs non-controlled) and type of intervention (HA vs HA plus CS) to ensure the robustness of the results. The findings of the present study indicate that HA/HA plus CS has a positive effect in the treatment of RUTIs. Indeed, given that there was a lack of information regarding prophylactic antibiotic use in many of the studies included, the positive effect of HA/HA plus CS found in this analysis is likely to be a conservative estimate. Definition of the primary outcome was usually clearly stated, but some outcomes were highly subjective and the retrospective registration in some studies may have introduced relevant observation bias. As suggested by the funnel plot analysis, reporting bias (particularly publication bias) is likely. On the other hand, the study populations were quite homogeneous in terms of inclusion and exclusion criteria, which may allow for generalization of the results.

The high heterogeneity among studies and the likelihood of publication bias are limitations that may lessen the validity and robustness of the results. Despite this, however, the qualitative and quantitative evaluation of the overall results are still considered to be useful and important. In fact, these data are of intrinsic interest, even if their interpretation should be approached with caution.

Another limitation of this review was the small number of studies included. Of the 8 studies used in the meta-analysis, only 2 were randomized controlled trials and only 1 of these was of double-blind design. The remaining 6 were nonrandomized studies, of which 4 were noncontrolled without a separate control group (only an internal control with a retrospective pre-treatment versus prospective post-treatment analysis). However, pooled data from all of these studies were used, and subgroup analyses were conducted on the basis of design (nonrandomized versus randomized and noncontrolled versus controlled studies). Among the studies considered in this meta-analysis, HA plus CS was the only product evaluated in randomized controlled trials, where the control group received standard prophylaxis for RUTIs (i.e., antibiotics) or placebo; therefore, these studies show the highest level of evidence. As HA alone was only investigated in noncontrolled studies with generally higher levels of bias, this evidence is considered to be of lower quality. Thus, differences in data quality, study design, and strength of evidence could bias the comparison between HA and HA plus CS. Of note, all studies included in this meta-analysis were conducted in Europe and, therefore, the generalizability of its results may be limited.

The results of the current meta-analysis are generally consistent with those of the previous meta-analysis published in 2013 [26], and provide an update to the earlier publication with the addition of two controlled studies [20, 24] and one large uncontrolled study [21] published from 2014 to 2016, and one abstract from 2011 [19].

## Conclusions

Intravesical instillation of HA, alone or in combination with CS, may be a promising and feasible treatment option for female patients with RUTIs and is generally well tolerated. This systematic review and meta-analysis has shown that HA or HA plus CS, compared with placebo, standard of care prophylaxis, or retrospective patient review, is associated with a decreased rate of UTI recurrence and with improved symptoms in adult females with a history of RUTI. This treatment may therefore offer an alternative to the widespread use of antibiotic prophylaxis. Given that antibiotic consumption is a driver for the development of antibiotic resistance [8, 9], and a report from the European Food Safety Authority/European Centre for Disease Prevention and Control states that both resistance and multidrug resistance are increasing, such that some agents are no longer appropriate treatments for some infections [36], alternatives to antibiotics are urgently needed. Since there are also increasing rates of antimicrobial resistance in pathogens that cause UTI [11, 14, 15], effective non-antibiotic-based treatments for these infections are also required.

It is noteworthy that evidence provided by randomized controlled trials was available only for HA plus CS. The quality of evidence in the studies on combination therapy was therefore higher than in those involving HA alone, although the limited number of available randomized controlled trials limits the ability to provide a definitive conclusion. Further research should evaluate the efficacy and safety of HA or HA plus CS compared with each other or with standard of care prophylaxis for prevention of RUTIs using well-designed, randomized, controlled clinical trials with larger patient populations.

## Electronic supplementary material

ESM 1 (DOC 1542 kb)
