# Supplementary material for: Intravesical hyaluronic acid and chondroitin sulfate for recurrent urinary tract infections: systematic review and meta-analysis
Source: Int Urogynecol J. 2017 Nov 27;29(7):933–42. doi: 10.1007/s00192-017-3508-z (PMC6004275; doi:10.1007/s00192-017-3508-z)

# Supplementary material

## Search strategy: MEDLINE (Pubmed)

1. "urinary tract infections"[MeSH]

2. urinary tract infections[TW]

3. Bacteriuria

4. bacilluria

5. "cystitis"[MeSH]

6. cystitis[TW]

7. Cystitides

8. #1 OR #2 OR #3 OR #4 OR #5 OR #6 OR #7

9. urinary

10. bladder

11. urine

12. urologic

13. ureteral

14. #9 OR #10 OR #11 OR #12 OR #13

15. infection

16. infections

17. inflammation

18. #15 OR #16 OR #17

19. #14 AND #18

20. pyelonephritis

21. urosepsis

22. uti

23. ruti

24. cauti

25. #20 OR #21 OR #22 OR #23 OR #24

26. #8 OR #19 OR #25

27. "hyaluronic acid"[MeSH]

28. hyaluronic acid[TW]

29. hyaluronic

30. hyaluronan

31. hyaluronate

32. "chondroitin sulfates"[MeSH]

33. #27 OR #28 OR #29 OR #30 OR #31 OR #32

34. chondroitin

35. condroitin

36. #34 OR #35

37. sulfate

38. sulphate

39. #37 OR #38

40. #36 AND #39

41. chondroitin*

42. "Cystistat"

43. "Gepan"

44. "Uracyst"

45. "Hyacist"

46. "Hyachon Duo"

47. "Instillamed"

48. #41 OR #42 OR #43 OR #44 OR #45 OR #46 OR #47

49. #33 OR #40 OR #48

50. "animals"[ MeSH]

51. "humans"[ MeSH]

52. #50 NOT #51

53. "male"[ MeSH]

54. "female"[ MeSH]

55. #53 NOT #54

56. "infant"[MeSH]

57. "child"[MeSH]

58. "infant, newborn"[MeSH]

59. "child, preschool"[MeSH]

60. #56 OR #57 OR #58 OR #59

61. "adolescent"[MeSH]

62. "adult"[MeSH]

63. "young adult"[MeSH]

64. "middle aged"[MeSH]

65. "aged"[MeSH]

66. "aged, 80 and over"[MeSH]

67. #61 OR #62 OR #63 OR #64 OR #65 OR #66

68. #60 NOT #67

69. #26 AND #49 NOT #52 NOT #55 NOT #68

## Search strategy: Embase

1. 'recurrent urinary tract infection'

2. 'urinary tract infection'/exp

3. 'infection, urinary tract'

4. 'lower urinary tract infection'

5. 'urinary infection'

6. 'urinary tract infection'

7. 'urinary tract infections'

8. 'urine infection'

9. 'urine tract infection'

10. 'urologic infection'

11. 'ruti'

12. 'cystitis'/exp

13. 'acute cystitis'

14. 'cystitis'

15. 'bacteriuria'/exp

16. 'bacilluria'

17. 'bacteriuria'

18. 'bacteruria'

19. 'urosepsis'/exp

20. 'urosepsis'

21. 'urological infection'

22. uti

23. cauti

24. #1 OR #2 OR #3 OR #4 OR #5 OR #6 OR #7 OR #8 OR #9 OR #10 OR #11 OR #12 OR #13 OR #14 OR #15 OR #16 OR #17 OR #18 OR #19 OR #20 OR #21 OR #22 OR #23

25. 'hyaluronic acid'/exp

26. hyaluronic

27. 'hyaluronate'/exp

28. 'hyaluronan'/exp 29. 'hyaluronan'

30. 'hyaluronate'

31. 'hyaluronate sodium'

32. 'hyaluronic acid'

33. 'hyaluronic acid component'

34. 'sodium hyaluronate'

35. 'chondroitin sulfate'/exp

36. 'chondroitin sodium sulfate'

37. 'chondroitin sodium sulphate'

38. 'chondroitin sulfate'

39. 'chondroitin sulfate sodium'

40. 'chondroitin sulfates'

41. 'chondroitin sulfuric acid'

42. 'chondroitin sulphate'

43. 'chondroitin sulphate sodium'

44. 'chondroitin sulphates'

45. 'chondroitine sulfate'

46. 'chondroitine sulphate'

47. 'chondroitinsulfate'

48. 'chondroitinsulfuric acid'

49. 'chondroitinsulphate'

50. 'chondroitinsulphuric acid'

51. 'Cystistat'

52. 'Gepan'

53. 'Uracyst'

54. 'Hyacist'

55. 'Hyachon Duo'

56. 'Instillamed'

57. #25 OR #26 OR #27 OR #28 OR #29 OR #30 OR #31 OR #32 OR #33 OR #34 OR #35 OR #36 OR #37 OR #38 OR #39 OR #40 OR #41 OR #42 OR #43 OR #44 OR #45 OR #46 OR #47 OR #48 OR #49 OR #50 OR #51 OR #52 OR #53 OR #54 OR #55 OR #56

58. [animals]/lim

59. [humans]/lim

60. #58 NOT #59

61. [male]/lim

62. [female]/lim

63. #61 NOT #62

64. [embryo]/lim

65. [fetus]/lim

66. [newborn]/lim

67. [infant]/lim

68. [child]/lim

69. [preschool]/lim

70. [school]/lim

71. #64 OR #65 OR #66 OR #67 OR #68 OR #69 OR #70

72. [adolescent]/lim

73. [young adult]/lim

74. [adult]/lim

75. [middle aged]/lim

76. [aged]/lim

77. [very elderly]/lim

78. #72 OR #73 OR #74 OR #75 OR #76 OR #77

79. #71 NOT #78

80. #24 AND #57 NOT #60 NOT #63 NOT #79

## Search strategy: Cochrane library

1. "urinary tract infections"[mesh]

2. Bacteriuria

3. bacilluria

4. "cystitis"[mesh]

5. Cystitides

6. #1 OR #2 OR #3 OR #4 OR #5

7. urinary

8. bladder

9. urine

10. urologic

11. ureteral

12. #7 OR #8 OR #9 OR #10 OR #11

13. infection

14. infections

15. inflammation

16. #13 OR #14 OR #15

17. #12 AND #16

18. pyelonephritis

19. urosepsis

20. uti

21. ruti

22. cauti

23. #18 OR #19 OR #20 OR #21 OR #22

24. #6 OR #17 OR #23

25. "hyaluronic acid"[mesh]

26. hyaluronic

27. hyaluronan

28. hyaluronate

29. "chondroitin sulfates"[mesh]

30. #25 OR #26 OR #27 OR #28 OR #29

31. chondroitin

32. condroitin

33. #31 OR #32

34. sulfate

35. sulphate

36. #34 OR #35

37. #33 AND #36

38. chondroitin*

39. "Cystistat"

40. "Gepan"

41. "Uracyst"

42. "Hyacist"

43. "Hyachon Duo"

44. "Instillamed"

45. #38 OR #39 OR #40 OR #41 OR #42 OR #43 OR #44

46. #24 AND #45

## Excluded studies

### Articles

1. Mañas A, Glaría L, Peña C, Sotoca A, Lanzós E, Fernandez C, Rivière M. Prevention of urinary tract infections in palliative radiation for vertebral metastasis and spinal compression: a pilot study in 71 patients. Int J Radiat Oncol Biol Phys. 2006;64(3):935-40.
2. Nordling J, van Ophoven A. Intravesical glycosaminoglycan replenishment with chondroitin sulphate in chronic forms of cystitis. A multi-national, multi-centre, prospective observational clinical trial. Arzneimittelforschung. 2008;58(7):328-35.
3. Raymond I, Vasdev N, Ferguson J, Haskin M, Davis L, Hasan TS. The clinical effectiveness of intravesical sodium hyaluronate (cystistat®) in patients with interstitial cystitis/painful bladder syndrome and recurrent urinary tract infections. Curr Urol. 2012;6(2):93-8.
4. Vedanayagam M, Brewin J, Briggs K, Salahia M.G, Hammadeh M.Y. The role of hyaluronic acid in the management of uncomplicated recurrent female urinary tract infections: Literature review and practical experience. Journal of Clinical Urology. 2013;6:4 (243-248).
5. Torella M, Schettino MT, Salvatore S, Serati M, De Franciscis P, Colacurci N. Intravesical therapy in recurrent cystitis: a multi-center experience. J Infect Chemother. 2013;19(5):920-5.
6. Costantini E, Lazzeri M, Pistolesi D, Del Zingaro M, Frumenzio E, Boni A, Pietropaolo A, Fragalà E, Porena M. Morphological changes of bladder mucosa in patients who underwent instillation with combined sodium hyaluronic acid-chondroitin sulphate (Ialuril®). Urol Int. 2013;91(1):81-8.
7. Ząbkowski T, Jurkiewicz B, Saracyn M. Treatment of Recurrent Bacterial Cystitis by Intravesical Instillations of Hyaluronic Acid. Urol J. 2015 1;12(3):2192-5.

### Abstracts

1. Gentile B.C, Giulianelli R, Albanesi L, Pisanti F. Intravesical administration of hyaluronic acid and choindroitin sulfate in prevention of recurrent bacterial cystitis: Preliminary study. Journal of Endourology. 2010;24 SUPPL. 1 (A204).
2. Sharifiaghdas F. Intravesical instillation of hyaluronic acid in the treatment of recurrent urinary tract infection in women. Urology. 2010;76:3 SUPPL. 1 (S102).
3. Fenner A. Infection: Intravesical hyaluronic acid and chondroitin reduces incidence of recurrent UTI. Nature Reviews Urology. 2011;8:4 (178).
4. Sommariva M, Sandri S.D. Bladder instillation of glucosaminoglicans (GAGS) and antibiotic in patients under intermittent catheterization and recurrent symptomatic urinary tract infections. Neurourology and Urodynamics. 2011;30 SUPPL. 1 (48-49).
5. Vasdev N, Ferguson J, Harding C, Dorkin T, Hasan T. The clinical effectiveness of intravesical cystistat® (Hyaluronic acid) in patients with refractory painful bladder syndrome or recurrent urinary tract infections. International Urogynecology Journal and Pelvic Floor Dysfunction. 2011;22 SUPPL. 2 (S1367).
6. Gentile B, Giulianelli R, Mirabile G, Mavilla L, Pisanti F, Albanesi L, Schettini M. Recurrent bacterial cystitis, bladder instillation with a combined solution of sodium halurate and chondroitin sulfate (IALURIL): One year of follow-up. Urology. 2011;78:3 SUPPL. 1 (S209).
7. Sakalis V.I, Giouris A, Gkotsi A, Papadopoulos A, Kangas A, Kipouros A. Preliminaty results from the endocystic administration of hyaluronic acid in female patients with recurrent lower urinary tract infections. European Urology. Supplements 2011;10:9 (588).
8. Gentile B.C.B.C, Giulianelli R.R, Schettini M.M, Pisanti F.F, Albanesi L.L. Recurrence bacterial cystitis: Bladder instillation with a combinated solution of sodium halurate and chondroitin sulphate (IALURIL). Journal of Endourology. 2011;25 SUPPL. 1 (A157).
9. Yoon H, Byun Y.J, Shim B.S, Chung W.S, Lee D.H, Park Y.Y. Early experience of safety and efficacy of sodium hyaluronatechondroitin sulfate solution in chronic painful conditions in the bladder. European Urology. Supplements 2013;12:1 (e512).
10. Hasan P, Perepanova T. The use of hyaluronic acid in chronic cystitis treatment. Urology. 2014;84:4 SUPPL. 1 (S242-S243).
11. Marri R, Housami F, Scott J, Lamb G. Intravesical sodium chondroitin sulphate in prevention of recurrent bacterial cystitis: Validation, efficacy and development of practice. Urology. 2014;84:4 SUPPL. 1 (S248).

# Supplementary tables

## Table S1. Risk of bias assessment for randomized studies, using the Cochrane risk of bias tool[^33^](#_ENREF_33)

| **Study** | **Adequate sequence generation** | **Allocation concealment** | **Blinding** | | **Description of losses and exclusions** | **Intention-to-treat analysis** | **Incompleteness of outcome data** |
| --- | --- | --- | --- | --- | --- | --- | --- |
|  |  |  | **Of participants and personnel** | **Of outcome assessors** |  |  |  |
| Damiano et al., 2011 | Yes | Yes | Yes | Yes | No | No | Unclear |
| De Vita et al., 2012 | Yes | Unclear | No | No | No | No | Unclear |

## Table S2. Risk of bias assessment for non-randomized studies, using the Risk Of Bias In Non-randomised Studies - of Interventions score[^34^](#_ENREF_34)

| **Study** | **Pre-intervention** | | **At intervention** | | **Post-intervention** | | |
| --- | --- | --- | --- | --- | --- | --- | --- |
|  | **Confounding bias** | **Selection bias** | **Classification bias** | **Deviation from intended interventions** | **Bias due to missing data** | **Bias in measurement of outcome** | **Selection of reported result** |
| Constantinides et al., 2004 | No | Unclear | Unclear | Unclear | Unclear | Yes | Unclear |
| Lipovac et al., 2007 | No | Unclear | Unclear | Unclear | Unclear | Yes | Unclear |
| Centemero et al., 2011 | No | Unclear | Unclear | Unclear | Unclear | Unclear | Yes |
| Cicione et al., 2014 | No | Unclear | Unclear | Unclear | Unclear | Unclear | Unclear |
| Gugliotta et al., 2015 | Unclear | Yes | Unclear | Unclear | Unclear | Yes | Unclear |
| Ciani et al., 2016 | Yes | Unclear | Unclear | Unclear | No | Unclear | Unclear |

## Table S3. Sensitivity analysis

| **Study removed from the meta-analysis** | **Outcome** | |
| --- | --- | --- |
|  | **Mean rate of UTI recurrence per patient-year, MD (95% CI)** | **Time to first UTI recurrence in days, MD (95% CI)** |
| None | –2.56 (–3.86, –1.26) | 130.05 (5.84, 254.26) |
| Constantinides et al., 2004 | –2.32 (–3.75, –0.89) | 77.4 (–3.99, 158.8) |
| Lipovac et al., 2007 | –2.25 (–3.61, –0.89) | 135.98 (–16.1, 288.06) |
| Damiano et al., 2011 | –2.4 (–3.89, –0.91) | 129.77 (–22.7, 282.24) |
| Centemero et al., 2011 | – | 126.07 (–26.32, 278.47) |
| De Vita et al., 2012 | –2.76 (–4.21, –1.3) | – |
| Cicione et al., 2014 | –2.37 (–3.84, –0.89) | 139.61 (–11.46, 290.69) |
| Gugliotta et al., 2015 | –2.87 (–4.23, –1.52) | – |
| Ciani et al., 2016 | –2.96 (–4.19, –1.73) | 172.34 (60.76, 283.93) |
| CI, confidence interval; MD, mean difference; UTI, urinary tract infection. | | |

# Supplementary figure legends

**Figure S1.** The effect of hyaluronic acid plus chondroitin sulfate versus control on (A) the number of 3-day voids and (B) 36-item Short-Form Health survey scores. CI, confidence interval; CS, chondroitin sulfate; HA, hyaluronic acid; RE, random effects; SD, standard deviation

**Figure S2.** The effect of hyaluronic acid or hyaluronic acid plus chondroitin sulfate versus on (A) mean UTI rate per patient-year and (B) time to first UTI recurrence (in days). Studies stratified by presence or absence of randomization. CI, confidence interval; CS, chondroitin sulfate; HA, hyaluronic acid; RE, random effects; SD, standard deviation

**Figure S3.** The effect of hyaluronic acid or hyaluronic acid plus chondroitin sulfate versus control on (A) mean UTI rate per patient-year and (B) time to first UTI recurrence (in days). Studies stratified by controlled/uncontrolled. CI, confidence interval; CS, chondroitin sulfate; HA, hyaluronic acid; RE, random effects; SD, standard deviation

**Figure S4.** The effect of hyaluronic acid or hyaluronic acid plus chondroitin sulfate versus control on (A) mean UTI rate per patient-year and (B) time to first UTI recurrence (in days). Studies stratified by type of active treatment. CI, confidence interval; CS, chondroitin sulfate; HA, hyaluronic acid; RE, random effects; SD, standard deviation

# Supplementary figures

## Figure S1.

(A)


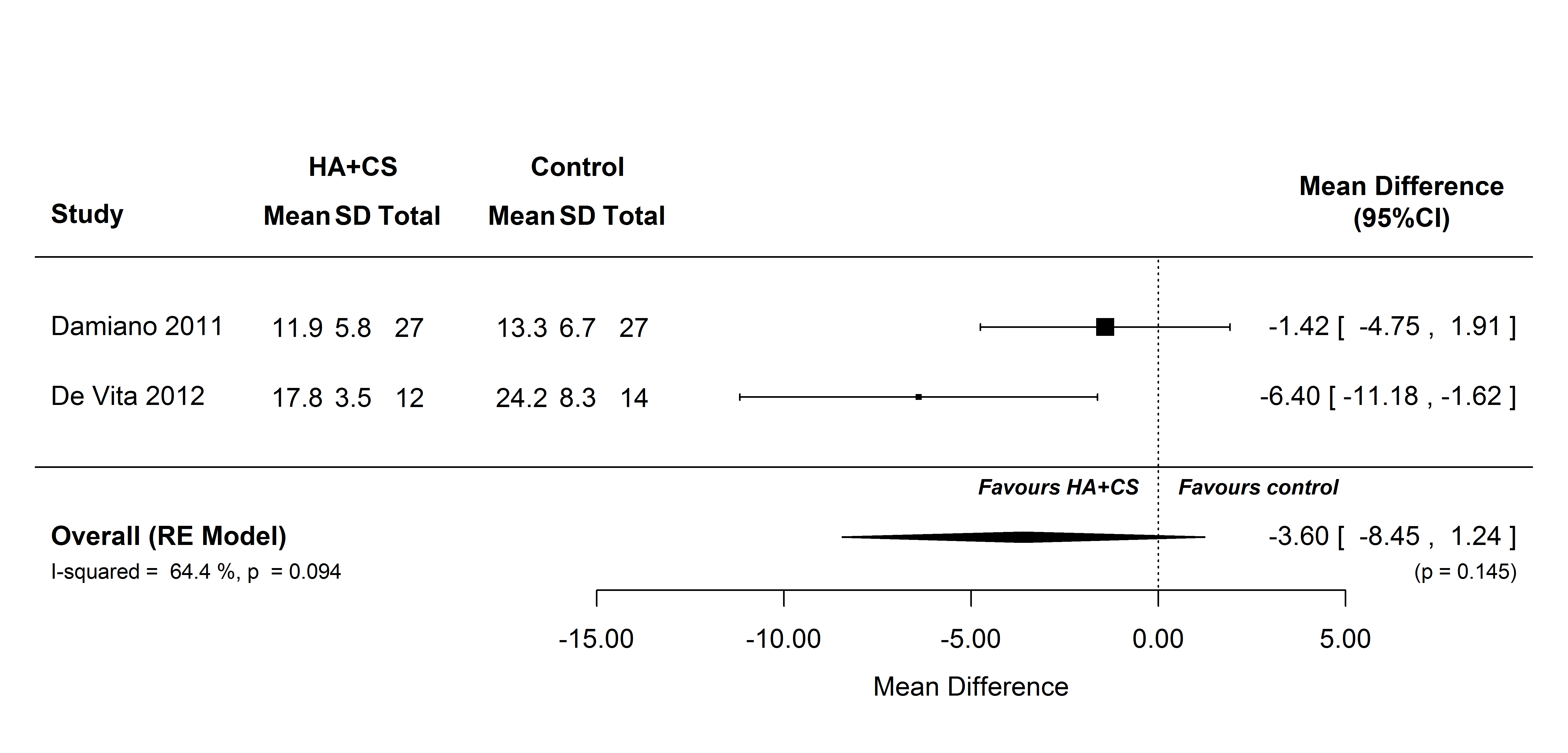


(B)


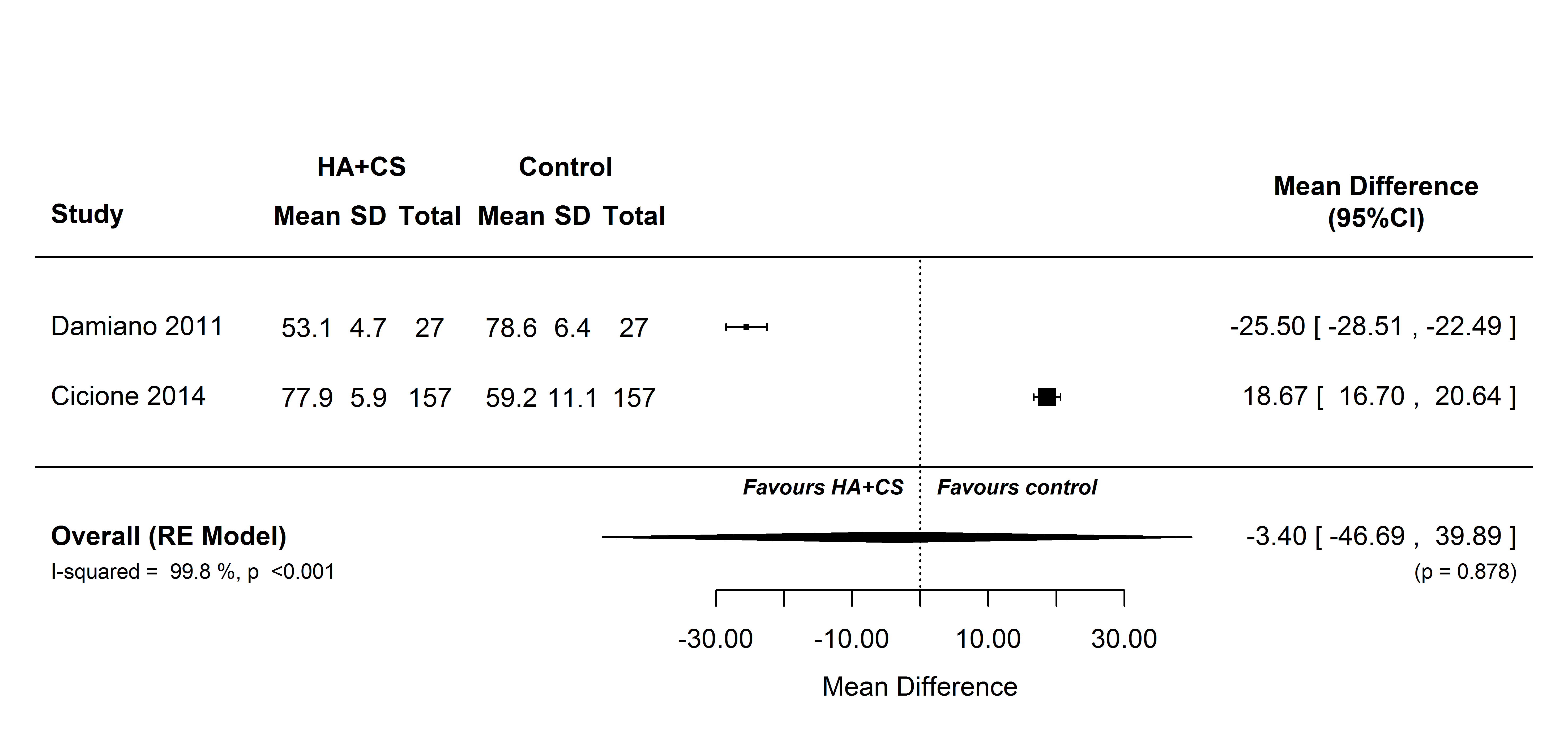


## Figure S2.

(A)


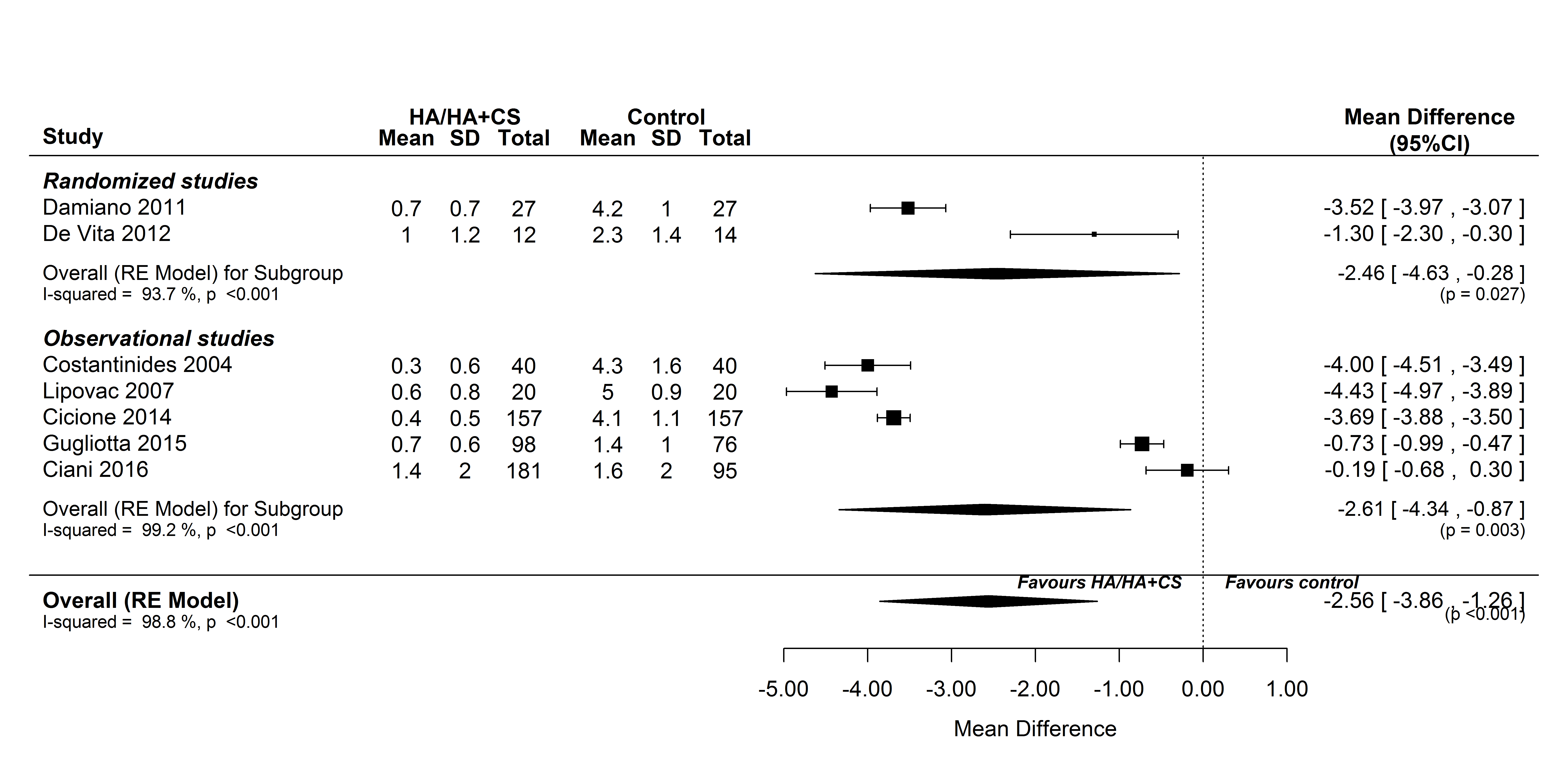


(B)


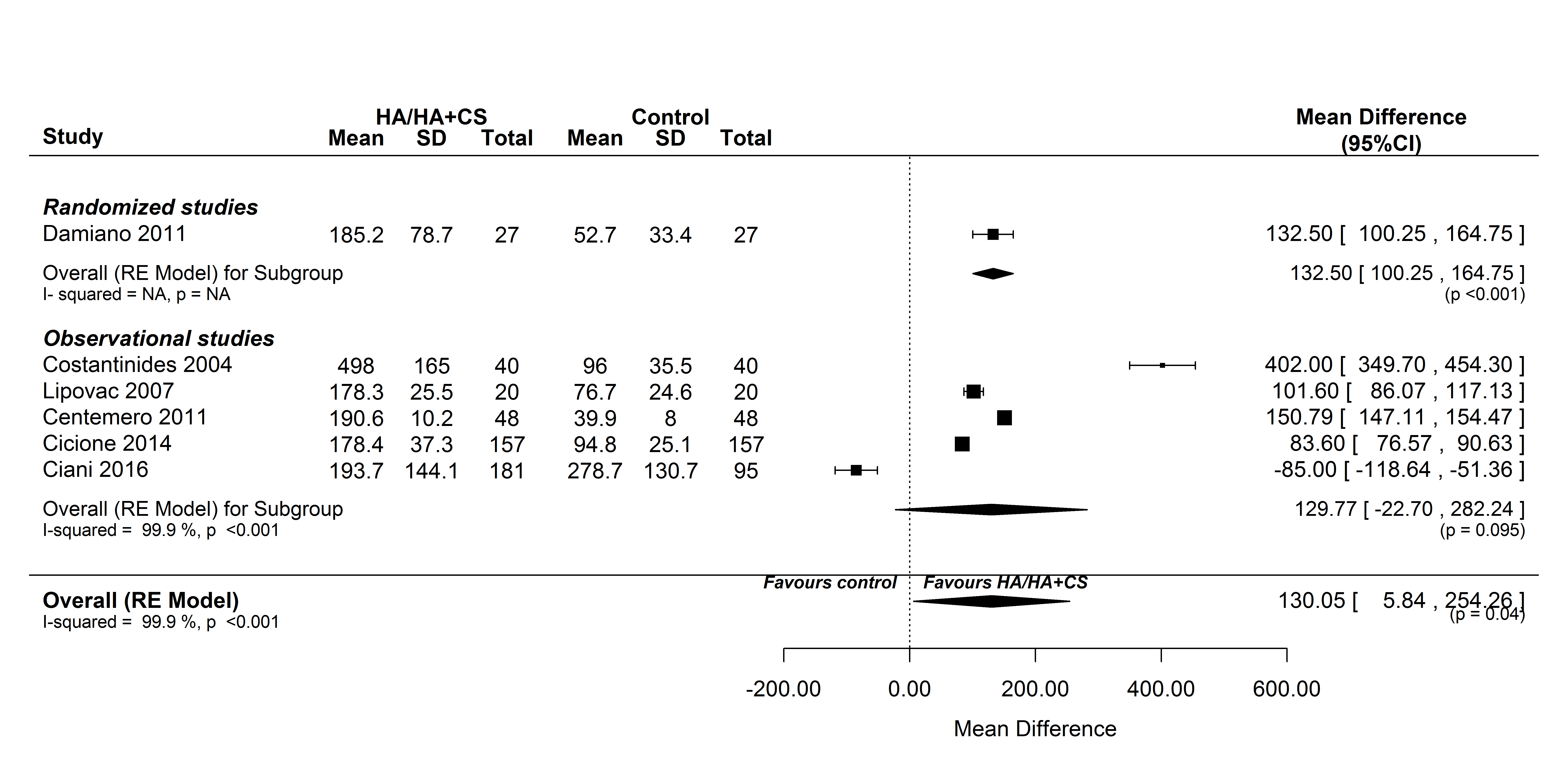


## Figure S3.

(A)


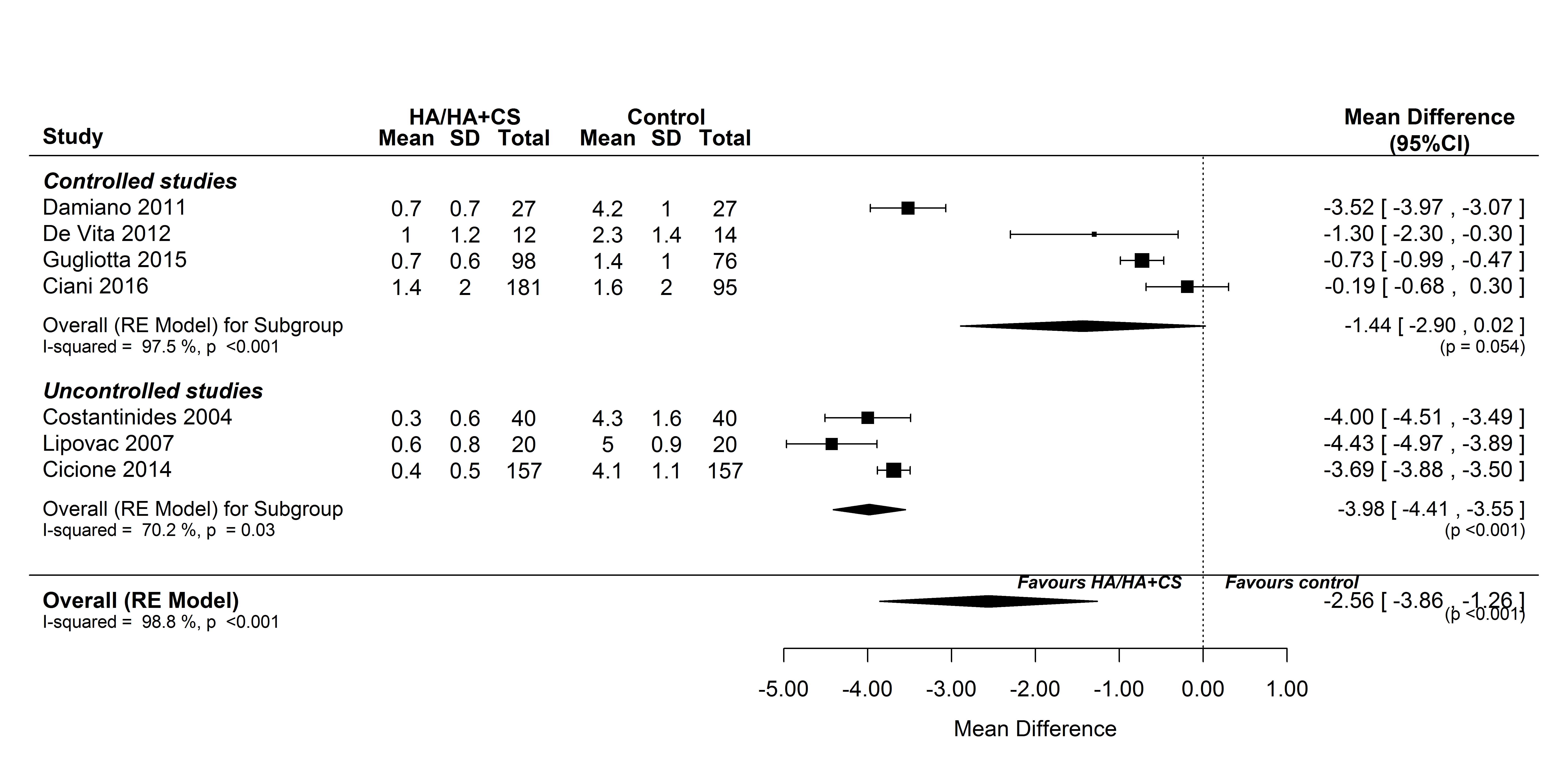


(B)


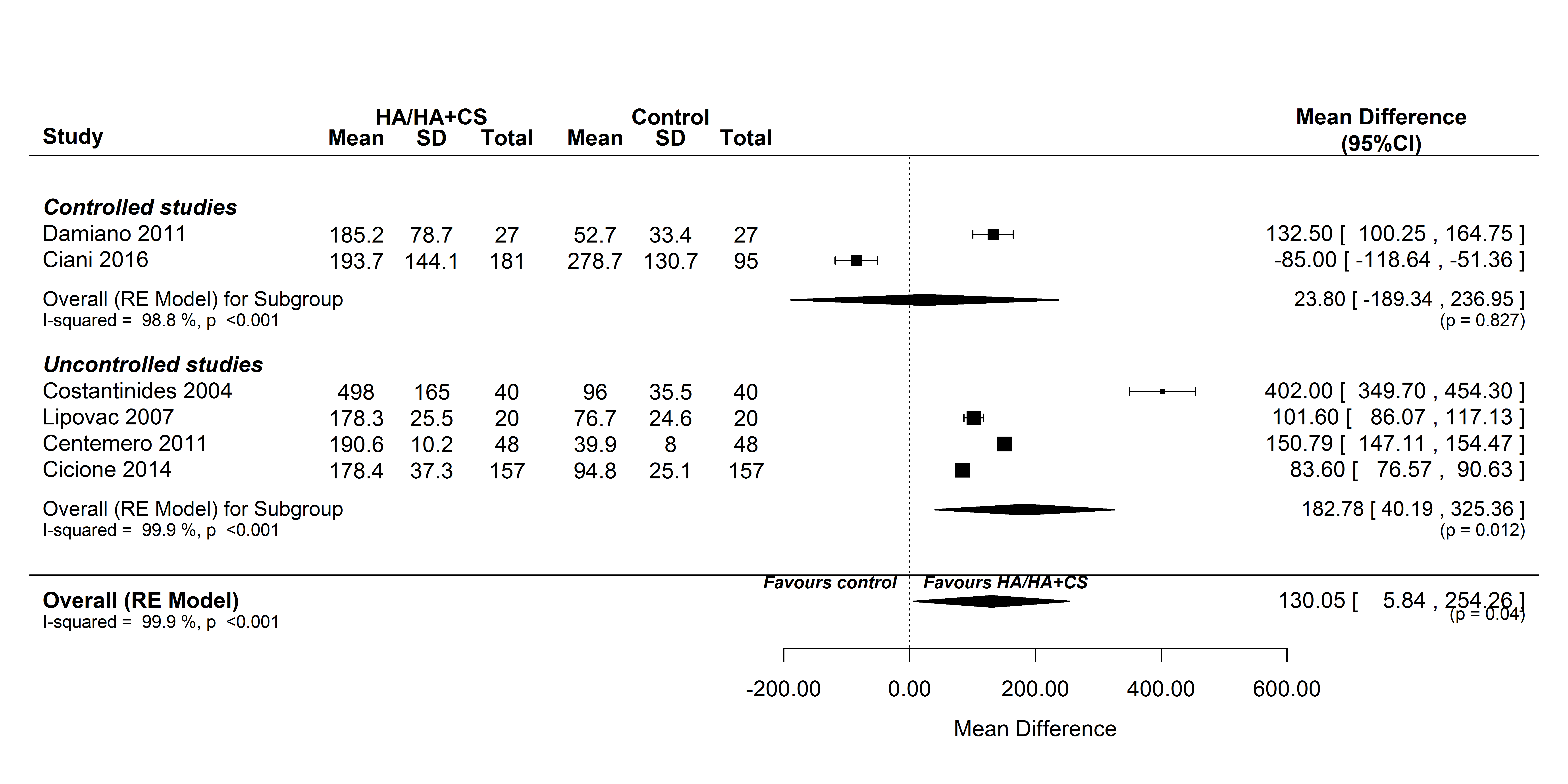


## Figure S4.

(A)


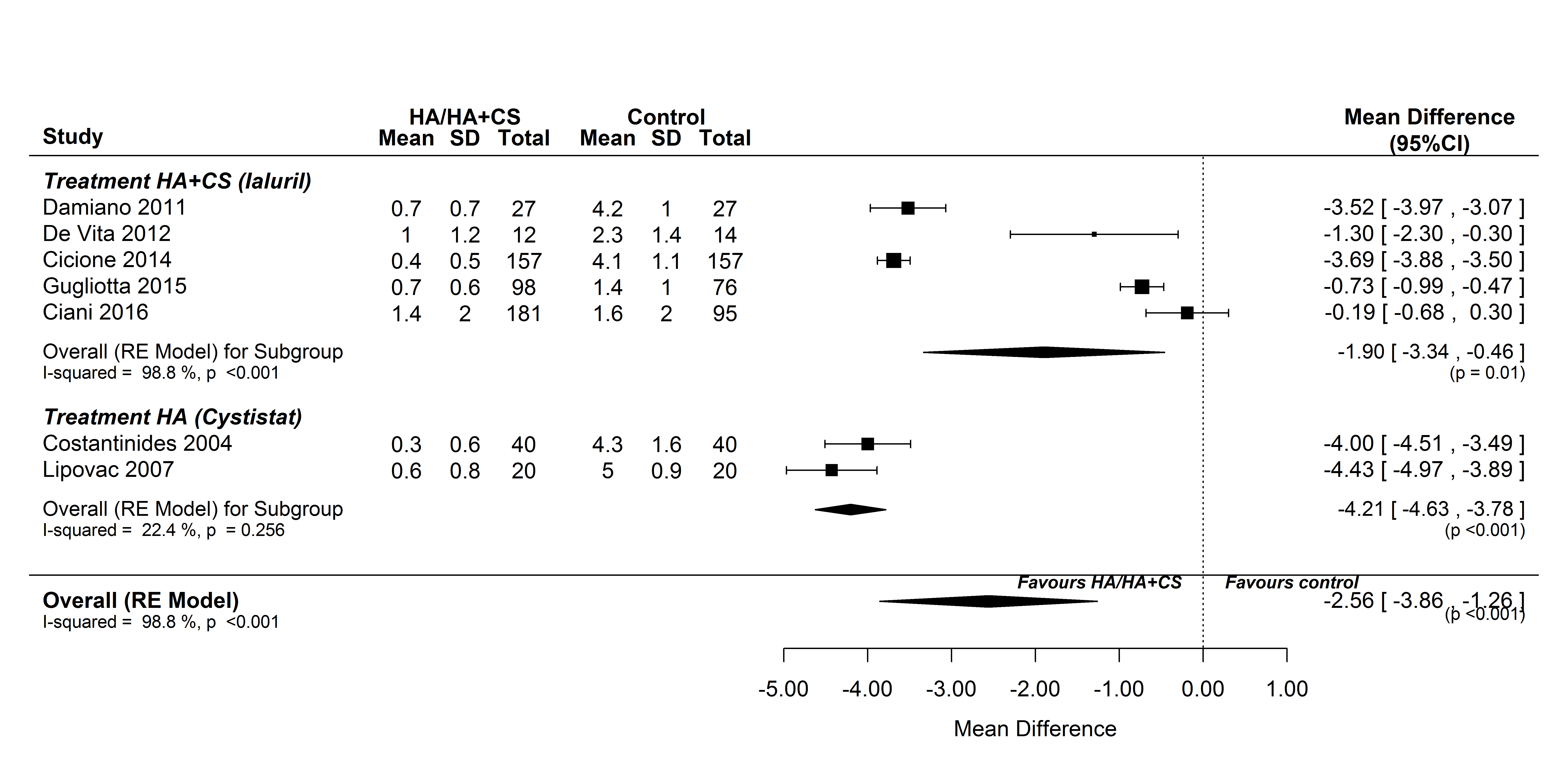


(B)


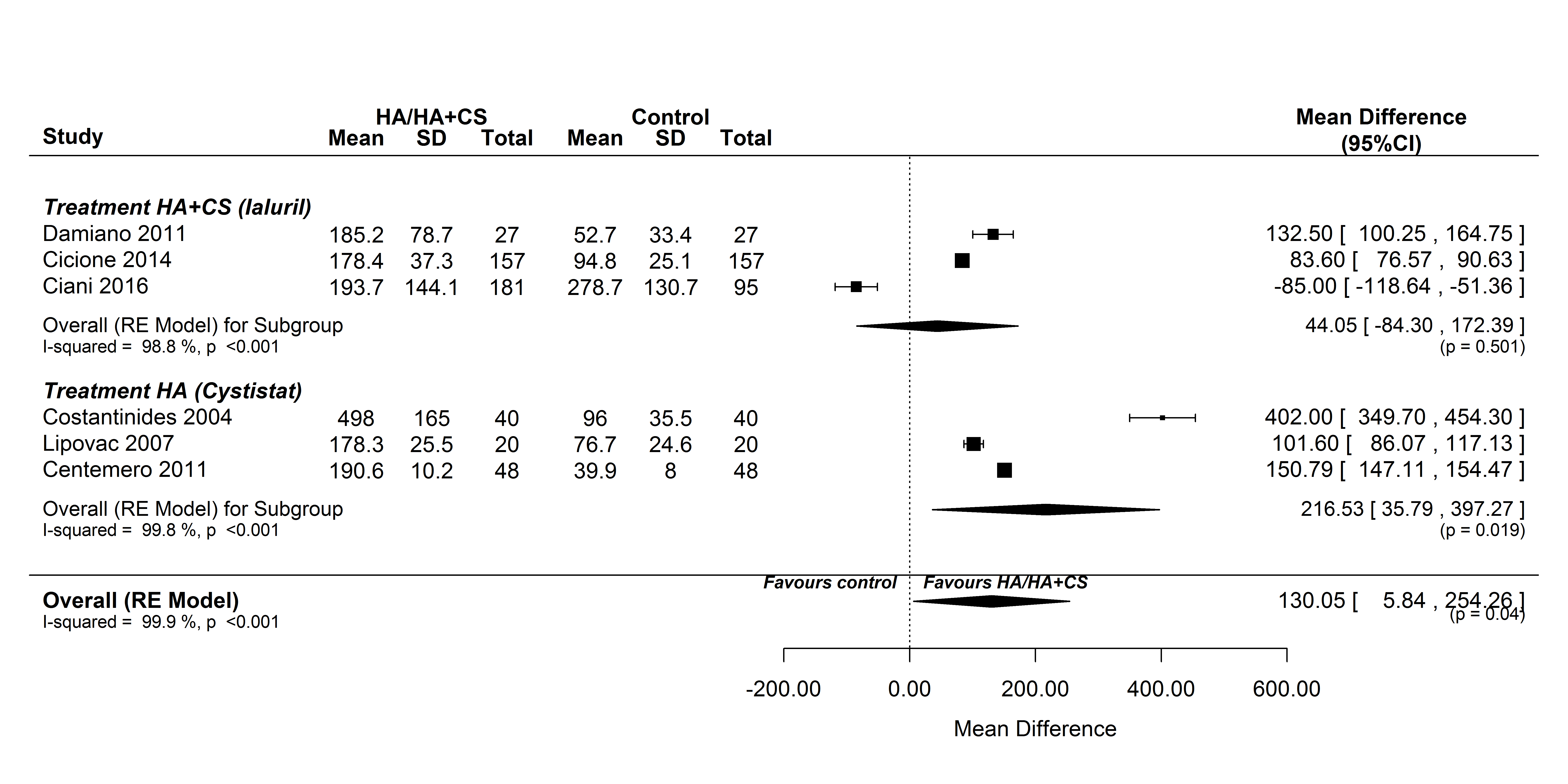

Supplement: Supplementary file 1 — (DOC 1542 kb) [file 192_2017_3508_MOESM1_ESM.doc]
